# Unsupervised versus Supervised Identification of Prognostic Factors in Patients with Localized Retroperitoneal Sarcoma: A Data Clustering and Mahalanobis Distance Approach

**DOI:** 10.1155/2018/2786163

**Published:** 2018-04-23

**Authors:** Rita De Sanctis, Alessandro Viganò, Alessandro Giuliani, Alessandro Gronchi, Antonino De Paoli, Pierina Navarria, Vittorio Quagliuolo, Armando Santoro, Alfredo Colosimo

**Affiliations:** ^1^Department of Medical Oncology and Hematology, Humanitas Cancer Center and Research Hospital, IRCCS, Rozzano, Milan, Italy; ^2^Molecular and Cellular Networks Lab, Department of Anatomy, Histology, Forensic Medicine and Orthopaedics, “Sapienza” University of Rome, Rome, Italy; ^3^Department of Neurology and Psychiatry, “Sapienza” University of Rome, Rome, Italy; ^4^Department of Environment and Health, Istituto Superiore di Sanità, Rome, Italy; ^5^Department of Surgery, Fondazione IRCCS Istituto Nazionale dei Tumori, Milan, Italy; ^6^Department of Radiation Oncology, Centro di Riferimento Oncologico, National Cancer Institute, Aviano, Italy; ^7^Department of Radiotherapy and Radiosurgery, Humanitas Cancer Center and Research Hospital, IRCCS, Rozzano, Milan, Italy; ^8^Department of Surgery, Humanitas Cancer Center and Research Hospital, IRCCS, Rozzano, Milan, Italy; ^9^Humanitas University, Rozzano, Milan, Italy

## Abstract

The aim of this report is to unveil specific prognostic factors for retroperitoneal sarcoma (RPS) patients by univariate and multivariate statistical techniques. A phase I-II study on localized RPS treated with high-dose ifosfamide and radiotherapy followed by surgery (ISG-STS 0303 protocol) demonstrated that chemo/radiotherapy was safe and increased the 3-year relapse-free survival (RFS) with respect to historical controls. Of 70 patients, twenty-six developed local, 10 distant, and 5 combined relapse. Median disease-free interval (DFI) was 29.47 months. According to a discriminant function analysis, DFI, histology, relapse pattern, and the first treatment approach at relapse had a statistically significant prognostic impact. Based on scientific literature and clinical expertise, clinicopathological data were analyzed using both a supervised and an unsupervised classification method to predict the prognosis, with similar sample sizes (66 and 65, resp., in casewise approach and 70 in mean-substitution one). This is the first attempt to predict patients' prognosis by means of multivariate statistics, and in this light, it looks noticable that (i) some clinical data have a well-defined prognostic value, (ii) the unsupervised model produced comparable results with respect to the supervised one, and (iii) the appropriate combination of both models appears fruitful and easily extensible to different clinical contexts.

## 1. Introduction

Retroperitoneal sarcomas (RPS) are a peculiar soft tissue sarcoma (STS) subgroup including 4 or 5 subtypes and an expected incidence of less than 1 case per 100,000 inhabitants/year [1–4]. Although surgery is the standard treatment for localized lesions [5–13], the role of radiation therapy (RT) in RPS is not fully defined as of yet. In order to complement surgery, the use of preoperative and intraoperative RT is under increasing investigation. In addition, chemotherapy (CT), concurrent to RT, could have a radiosensitizer effect and a precautionary role in eradicating micrometastases, thus increasing the therapeutic index of RT alone [14, 15].

The analysis of prognostic factors and risk stratification, in order to decide the better therapeutic approach, is still pivotal in patients with rare tumors; moreover, the usefulness of the few tools available to predict good or poor prognosis is not completely defined [16].

In this contribution, we present an exploratory univariate analysis of possible disease predictors as well as a multivariate prognostic model for RPS based on discriminant function analysis (DFA), Mahalanobis distance (MD), and decision trees (DT) classification. An unsupervised approach in the analytical strategy showed a comparable efficiency with respect to a supervised one, which is suggestive of their possible combined use in clinical practice.

## 2. Materials and Methods

### 2.1. Clinical Data

Clinicopathological data about RPS were obtained from patients treated at three Italian referral centers according to the protocol Italian Sarcoma Group-Soft Tissue Sarcoma Trial 0303 (ISG-STS0303; EudraCT number: ITASARC_^*∗*^II_2004_003) between December 2003 and December 2010. At the end of this protocol, patients underwent further follow-up, thus collecting further information concerning (i) pattern of relapse (local and/or distant), (ii) disease-free interval (DFI), and (iii) postrelapse outcome (type of treatment at recurrence, response rate according to RECIST (Response Evaluation Criteria in Solid Tumors) criteria [17], and postrelapse survival).

Institutional databases from the 3 main participating enrolling centers (Fondazione IRCCS Istituto Nazionale dei Tumori, Milan, Italy; Humanitas Research Hospital, IRCCS, Rozzano, Milan, Italy; and CRO, IRCCS, Aviano, Italy) allowed updating the follow-up of 70 patients operated on with a median follow-up time from surgery of 91.7 months (interquartile range (IQR): 72.5, 111.3 months). Patients lost to follow-up were excluded from the analysis. Male/female ratio was 1.25 : 1. Median age was 58.5 years (range: 27–75). The patients' distribution among six relevant clinical descriptors is reported in Table 1.

Notice that, for the 70 patients considered in Table 1, the following information was also available: type of resection (macroscopically complete or incomplete, multivisceral surgery), adjuvant therapies, percentage of necrosis of the surgical specimen, DFI, relapse pattern (local and/or distant), first type of treatment at recurrence (first-line chemotherapy regimen, best response and RT), and further treatments (second-line chemotherapy regimen, best response, and further CT lines).

41 out of the 70 patients included in the study developed local (*n* = 26) or distant (*n* = 10) or both local and distant (*n* = 5) relapse. Main histological relapsed subtypes were dedifferentiated liposarcoma (14 out of 41, 34%), leiomyosarcoma (10, 24%), well-differentiated liposarcoma (6, 14%), and NOS (not otherwise specified) sarcoma (6, 14%). Median DFI was 29.47 months. Patients with any local relapse (with or without distant disease) presented an infield (23/31), outfield (4/31), or mixed (4/31) recurrence. Among patients with only local relapse, 16/26 (61.5%) received surgery (in two cases after preoperative chemotherapy), 9/26 (34%) received chemotherapy, and 1 (4%) received RT. After metastatic relapse, 2/15 (13%) patients underwent surgery, 9/15 (60%) first-line chemotherapy, and 4/15 (27%) both. In addition, four (27%) of these patients received palliative RT. Of the 41 relapsed patients, sixteen (39%) and 4 (9.7%) received ≥2 and ≥3 lines of chemotherapy, respectively. The most commonly used agents included anthracyclines, trabectedin, and gemcitabine. The response rate to first-line chemotherapy was 23% and 10% to subsequent lines.

**Figure 1:**
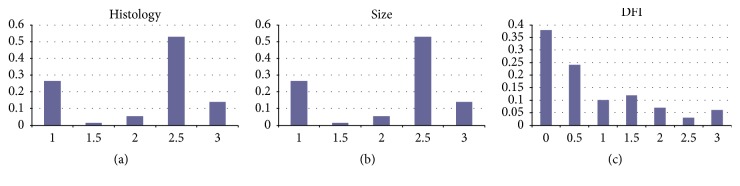
*Distribution of histology (a), size (b), and disease-free interval or DFI (c) among the patients considered in this work, according to Table 2, recoding parameters.* In histology (a), group 1 indicates WDLPS and SFT, group 1.5 myxoid LPS, group 2 pleomorphic LPS and fibrosarcoma, group 2.5 LMS and DDLPS, and group 3 MPNST and NOS sarcoma. In size (b), group 1 (<10 cm), group 1.5 (11–15 cm), group 2 (16–20 cm), group 2.5 (20–30 cm), and group 3 (30–45 cm) correspond to different sizes in the largest diameter of the tumor. In DFI (c), group 0 corresponds to never relapsed patients, groups 0.5 stays for patients with a DFI superior to 36 months, group 1 is for a DFI from 24 to 36 months, group 1.5 is for a DFI ranging from 12 to 24 months, group 2 is for a DFI of 6–12 months, group 2.5 is for a DFI inferior to 6 months, and group 3 is for patients with no eradication of the disease (never NED). Notice that (i) liposarcoma and leiomyosarcoma accounted for 75% of all patients, (ii) the variable size was normally distributed, and (iii) the majority of patients treated on the protocol ISG-STS 03.03 were in “no relapse” (0 group), confirming the potential effectiveness of the therapeutic approach.

In order to test the significance of any statistical model based upon the various diagnostic–therapeutic–prognostic parameters arising from clinical practice, a first and most important step was the recoding of those parameters on homogeneous scales so that quantitative classification and comparison become possible. An exemplary application of the above is illustrated in Figure 1, where, with the aim of checking a therapeutic approach, three clinical parameters, namely, DFI, tumor size, and histology, were numerically scaled by a 4-point (0–3) rating scale, in which 0 corresponded to a null risk and 3 to the highest risk of poor prognosis (see Table 2).

### 2.2. Statistical Methods

#### 2.2.1. Setup of a Data (Cases/Variables) Matrix

From the available clinicopathological information, some relevant variables endowed with good and similar prognostic value were selected and tested in order to optimize their prognostic value. Chemotherapy regimens and second-line therapies as effective modifiers and potential confounders, respectively, were eliminated. As a matter of fact, different chemotherapeutic agents, potentially active in specific histologies, may contribute to a different prognosis of the patients' outcome. On the other hand, second-line therapies could have an uncertain impact on the outcome: a patient undergoing a further chemotherapy line should live a sufficient time to undergo a second-line therapy, but second-line therapies are offered to progressive patients with evidence of disease.

Variables a priori considered as possible confounders of the exposure–outcome association(s) and also possible modifiers of the size or even the direction of the association between exposure and outcome were filtered out. Thus, the resulting data set included 9 main variables for each patient. If necessary, relevant data were numerically recorded and reclassified in groups at a 0.5-unit resolution (for the rescale parameters, see Table 2). These groups ranged from 0 to 3, where 0 corresponded to the better outcome while 3 corresponded to the worst prognosis. As an example, for the first type of retreatment at recurrence, an adequate treatment comprising radical surgery with or without radiotherapy was considered as the best approach (and reclassified as 1) in the prognosis, while a debulking surgery, which is known to be detrimental in the management of retroperitoneal sarcomas, was considered as the worst possible therapeutic approach (and, therefore, it was reclassified as 3).

#### 2.2.2. Data Analysis (Univariate and Multivariate Methods)

The association of the survival status with clinical variables was analyzed by univariate analysis, and the multivariate analysis was conducted at different levels of supervision applied to the independent variables. On the basis of the scientific literature and of clinical expertise of specialists in the field, the multivariate approach focused on the supervised clustering by discriminant function analysis (DFA) of clinicopathological profiles endowed with similar prognostic impact and Mahalanobis distance. It is worth stressing the particular meaning we associate here with the term “supervised.” On a purely statistical (syntactic) perspective, a discriminant analysis is by definition a supervised approach, even if we do not attach an a priori weight to the intervening variables, given that the system optimizes the fitting to a known outcome. Here, we adopt a “semantic” definition of the term implying the a priori setting of the “weight of evidence” of each variable, instead of limiting ourselves to make this weight emerge a posteriori by the least square optimization. We could use the term “Bayesian” for this approach, but we prefer “supervised” given that Bayesian approaches imply a particular mathematical computation of a posteriori probabilities that we do not apply here.

DFA estimates the linear combinations of descriptors maximizing the separability among subjects according to their survival status [18]. DFA and Mahalanobis distance classification was performed on the clinical regressors (previous medical knowledge and data fitting). DFA allowed building a model able to predict the group (alive/dead) that each patient belongs to, through a forward stepwise optimization paradigm. Structural classification was derived from the whole dataset of variables to see if individuals could be grouped into any natural system of groups.

The Mahalanobis distance is a measure of the distance between a point *P* and a distribution *D* introduced by Mahalanobis in 1936 [19]. It is a generalization of the Euclidean distance taking into consideration the mutual empirical correlation allowing the estimation of the distance of a unit (in our case a patient) from his/her reference population. This distance is zero if *P* is at the mean of its group (being the mean defined as a vector of *k* components correspondent to the means of the *k* variables) and grows as *P* moves away from the mean. In the case of two-class discrimination, the Mahalanobis distance of a patient *P* from the centers of the two classes is computed and *P* assigned to the nearest group.

The main outcome of univariate and multivariate analyses was the assignment of each patient to one of the two alive/dead groups, and results were considered significant at *p* < 0.05, after correction.

The statistical analyses were carried out by JMP version 13 and STATISTICA version 7.

## 3. Results

### 3.1. Univariate Analysis

Taking the vital status of patients as the main outcome in the univariate analysis, the association of the outcome (survival status) with each clinical variable of interest was examined, regardless of all other variables. We found that histology, grading, response to preoperative treatment, disease-free interval (DFI), pattern of relapse, and first treatment approach at relapse showed a relatively better prognostic impact (Table 3). Notice that, in spite of the relatively fewer cases considered in the unfiltered condition, the overall association performance of the abovementioned variables was not improved in the “filtered” homogeneous condition, which was entitled to focus on the latter in the following analyses.

### 3.2. Multivariate Analysis

Since the multivariate version of DFA is particularly sensitive to the number of cases, we recovered as many cases as possible by both “mean-substitution” and “casewise” validation procedures. In the mean-substitution procedure, missing data were substituted by their respective means. In the casewise method, those patients with too many missing values were automatically excluded from the analysis: out of 9 patients with missing values (Table 4), 4 and 5 patients were excluded from the supervised and unsupervised multivariate analysis, respectively. Under both conditions, variables were stepwise added to the discriminant function with an Enter *F* of 1.00, an Exit *F* of 0.0, and a tolerance of 0, through a forward stepwise method, selecting at each step the variable that made the most significant contribution to the discrimination.

Casewise DFA (*n* = 66 patients) showed that DFI, age, and histology were endowed with a relevant predictive value, while the mean-substitution (*n* = 70 patients) method found DFI, relapse pattern, and histology as the best predictors. In both cases, DFI and histology were the most and the least important variables, respectively (see Table 5). For each variable, Wilks' lambda and its *p* value have been reported. Wilks' lambda is a statistical test used in multivariate analysis of variance (like a *t*-test in the multivariate setting) to test whether there are differences between the means of the samples on a combination of dependent variables. In the present case,* Wilks' lambda* indicated the unique contribution of the respective variable to the discrimination.

### 3.3. Multivariate Analysis: Supervised and Unsupervised Methods

In the unsupervised analysis, the rescaling of each variable was not performed and, most importantly, the variables were not a priori selected according to clinical expertise or literature data. All the known and independent variables were possible candidates for inclusion into the model. Under these conditions, the 16 variables initially found independent of each another (gender, histology, grading, tumor size, response to neoadjuvant CT-RT, multivisceral surgery, adjuvant therapies, margins, DFI, relapse, local recurrence, distant metastases, surgery for relapsed disease, first-line CT, and second-line CT) were submitted to the further constraint of the minimum possible number of missing values and finally reduced to 9.

In the unsupervised analysis, the discriminant function showed that DFI had a statistically significant predictive value in both casewise (*n* = 65 patients) and mean-substitution (*n* = 70 patients) methods (Table 5).

### 3.4. Canonical Analysis

**Figure 2:**
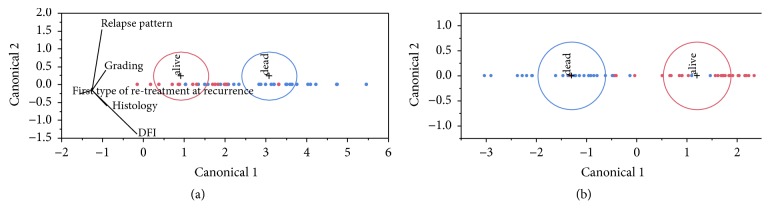
*Scatterplots in monodimensional canonical space*. (a) Supervised condition (cases # = 64 (dead = 23, alive = 30); wrong = 11; % wrong = 17.2; *R*^2^ = 0.51). (b) Unsupervised condition (cases # = 56 (dead = 25, alive = 25); wrong = 6; % wrong = 10.7; *R*^2^ = 0.55).

A canonical analysis is generally used to get some orthogonal (independent of each other) discriminant functions through the computation of orthogonal discriminant roots. Computationally, a canonical correlation analysis determines the successive discriminant functions and canonical roots, corresponding to the eigenvalues associated with the respective canonical function. The maximum number of functions cannot exceed the number of groups used in the classification minus one. Thus, in the present case, one discriminant function was estimated, which provided the best overall discrimination between alive and dead patients: in Figures 2(a) and 2(b), alive patients (red dots) are concentrated in the left or the right part, respectively, of the scatterplot.

Since the main goal of any DFA method is to classify cases, a distance estimator in the multivariate space defined by the model's variables is needed. In such a context, Mahalanobis distance has been selected since it takes into account the existing correlation structure of the data, generating a well-conditioned metric for the system at hand. In brief, points are classified as belonging to distinct groups by measuring the distance of each point *P* from the multidimensional mean (centroid) of a distribution according to the covariance of the same distribution, so to scale the actual Euclidean distance in the multivariate space by the mutual correlation of the intervening variables. Briefly, such distances can be considered as weighted Euclidean distances; the model derived from a representative sample of the population at hand could be easily extended and applied to new clinical cases. If the distance from the centroid is higher than a certain threshold, the point is no longer considered as belonging to the group.

The classification method based on Mahalanobis distance showed in the unsupervised method 86.6% and 83.3% of accuracy of prediction of patients' outcome for casewise and mean-substitution methods, respectively (Table 6, columns 4 and 5), and in the supervised method 85.3% and 84.7% accuracy of prediction between alive and dead patients in casewise and mean-substitution method, respectively (Table 6, columns 2 and 3). Such accuracy values are quite high, even if post hoc classifications are considered.

### 3.5. Partitioning Classes (Groups) by Decision Trees Methods

**Figure 3:**
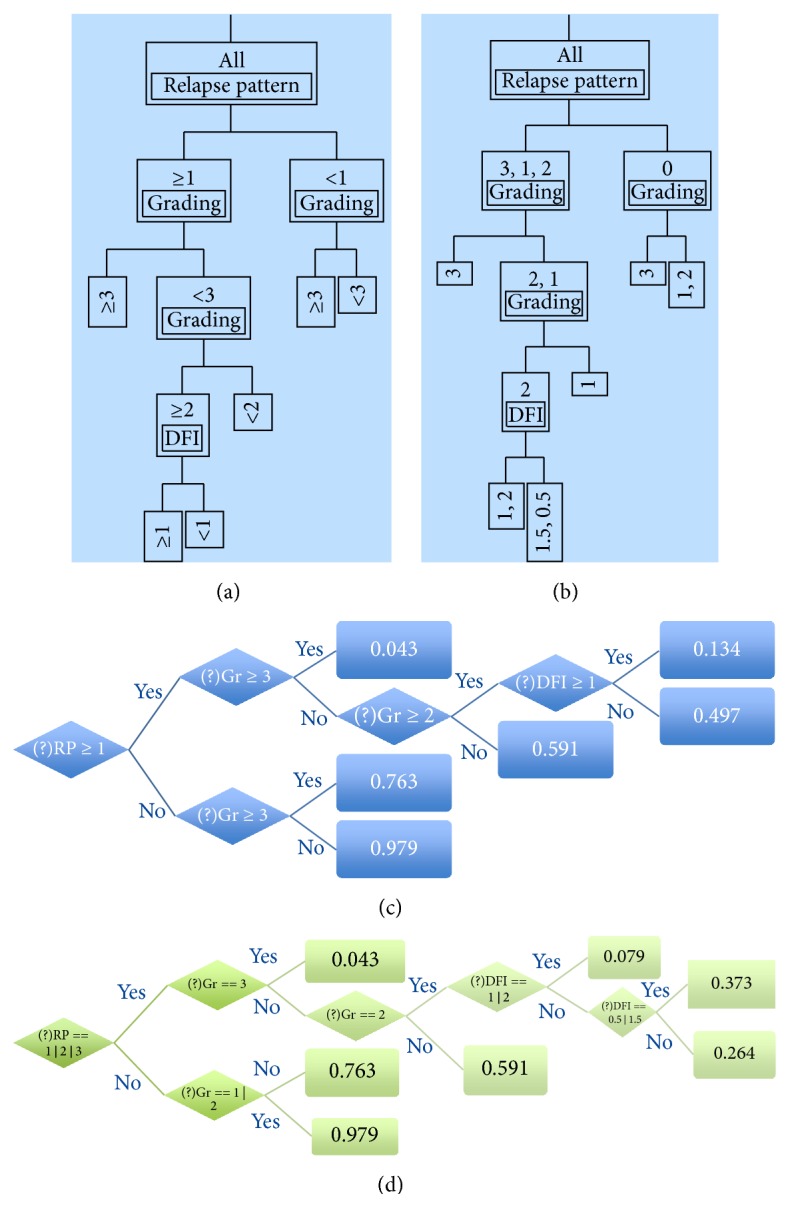
*Decision tree picture of the supervised- and unsupervised-based partitioning*. Panels (a) and (c) depict the first three branches (splits) of the decision tree obtained by the numeric, supervised coding (scales reported in Table 2) of the 5 best performing variables in Table 3 (histology, grading, DFI, relapse pattern, and 1st-type treatment at recurrence). Panels (b) and (d) refer to the same data coded as alphanumeric symbols, hence loosing any quantitative specificity assigned by supervisors. The rectangular boxes in panels (c) and (d) contain the *R*^2^ values, namely, an indication of the % of explained variability. Ideally, repeated partitioning should eventually produce a total *R*^2^ = 1. Modeling has been carried out by the Partition Platform of JMP, version 13.

Partitioning multivariate data according to a relationship between the predictors and response values creates an empirical decision tree useful for exploring relationships in the absence of a good prior model.  Figure 3 shows how a decision tree can visually and explicitly represent our database in a typical diagnostic or prognostic context.

A classic application of partitioning is to create a diagnostic heuristic for a disease. Moreover, given symptoms and outcomes for a number of subjects, partitioning can be used to generate a hierarchy of questions helpful for new patients' prognosis. Figures 3(a) and 3(b) depict the first three partitions (decision trees) identifying the probable alive and dead subjects on the basis of five selected variables (see the legend to the figure). Such variables were coded by numeric and alphanumeric symbols, in order to reproduce supervised and unsupervised learning, respectively. The diagrams in Figures 3(c) and 3(d) correspond to Figures 3(a) and 3(b), respectively. In spite of the intimidating aspect, such diagrams are a direct application of simple logical rules for the probable association of each single subject with the “alive” or dead “group” on the basis of the predictor variables, and they actually open the door to the prognostic application of the model to new subjects. It is important to note, however, that enriching the currently available database in order to check the model by the traditional approach of splitting the cases into two groups (to be used in a “learning” and a “test” phase) is in due course now.

## 4. Discussion

Recently, concomitant chemo- and radiotherapy proved to be a safe and promising treatment in RPS, leading to an increase of the 3-year relapse-free survival (3y-RFS) with respect to historical controls. However, no significant prognostic factors were found by the classical Cox proportional hazards model, except for a trend toward a better RFS and overall survival (OS) in patients with nonliposarcoma histology [14]. To date, only nomogram-based prognostic tools are available for RPS [16], and thus the present study is, to our knowledge, the first application of univariate and multivariate methods to the prediction of clinical outcome in STS patients.

In univariate analysis, several variables, chosen among the more important clinicopathological parameters, showed a significant prognostic role in the cohort of RPS patients treated on ISG-STS 03.03: DFI and relapse patterns appeared to be significantly associated with survival status, while size and age failed to reach statistical significance. The lack of significance of age is probably due to the epidemiology of the disease, mainly affecting young adults, and the inefficacy of size may be attributed to its anatomical peculiarity. The retroperitoneal site in fact, since it is not always directly in contact with abdominal organs, requires a highly specialized surgery which may limit the local invasiveness of the disease.

The effectiveness of the multivariate approach was tested in the context of both a supervised and an unsupervised method. Since, according to several sources [20, 21], DFA is very sensitive to outliers, we carried out the supervised analysis first, in order to reduce the variance of variables distribution and the number of outliers.

In the supervised classification model for DFA, the input variables were chosen and stratified by physicians on the basis of their clinical expertise in identifying lower and higher risk classes correlated with a proportional risk of death. Thus, the “relapse pattern” variable was defined as 0 if the patient did not experience a recurrence, 1 if the patient developed a local relapse, 2 if metastases were detected, and 3 if both local and distant relapse were diagnosed. Quite interestingly, the longest DFI, classified as 0, or null risk of progression and death, showed a statistically significantly positive prognostic role. In addition, in the supervised method, the gender was not included since, at present, any suggestion of a prognostic role of the gender in STS is missing and, finally, surgical margins (R0, R1, and R2) and relapse (yes/no) were reported in a more synthetic form.

In the unsupervised model, we introduced in the model all relevant variables without any a priori selection or rescaling, but with the lowest minimum possible number of missing values. Since every patient with missing values should be excluded from the analysis and, at the same time, a very limited sample size would thwart any statistics, 8 of the initial 24 variables, biased by several missing values, were excluded from the analysis.

In both supervised and unsupervised learning methods, we carried out the DFA analysis following either the* mean-substitution* or the* casewise* procedure. The results of the supervised and unsupervised model were fairly similar, with a misclassification rate of 15.3% and 16.7% when missing values were substituted by their respective means, and 14.7% versus 13.4% in the casewise setting, respectively. However, in some cases, the substitution of the missing values by the means could be inappropriate, such as for histology or response to preoperative chemoradiotherapy.

In the supervised model, DFI and histology were the best predictors in both casewise and mean-substitution approaches. Age was significant in casewise analysis and relapse pattern was significant in mean substitution. Indeed, the main factors influencing patients' outcomes are expected to be grading, with a poorer prognosis for G3 histologies, and histotype, with a greater metastatic potential for leiomyosarcomas and MPNST.

The role of DFI as a predictor of outcome seems straightforward since a longer time interval to relapse is intuitively a good prognostic factor and it could include other variables by itself, such as adequate surgery, response to preoperative treatments, or a less aggressive disease. It is worth noticing that, in the unsupervised model, only DFI was significant in both casewise and mean-substitution approaches.

Concerning the results presented in Figure 3 on the decision tree methods, the following points deserve attention.

(1) The *R*^2^ quantitative indicators of the obtained stratification are the same in the supervised and unsupervised procedure. In spite of the small number of clinically homogeneous subjects which could impair the stability of our statistical model, the emerging indication is that the two approaches are not incompatible among each other. A necessary prerequisite for an optimal diagnostic/prognostic performance remains, in any case, a solid expertise in the appropriate management of (possibly both, but at least or) clinical or statistical information.

(2) The diagrams of the type in panels (c) and (d), once properly mastered, may play a crucial role in extending the decision tree method to any clinical condition of similar or different pathology.

(3) On a more theoretical ground, it is worth noting that, in the multivariate approach (both supervised and unsupervised), we rely on “configuration of symptoms” and not on the simple additivity of single prognostic factors. This is particularly evident for Mahalanobis distances where the classification comes up from the distance computed on the whole *k*-dimensional space. This implies that even information that per se does not have a relevant prognostic power can contribute to the classification when considered altogether.

## 5. Conclusions

All in all, it seems fair to conclude that unsupervised and supervised analyses produced slightly similar results and a fair outcome prediction in retroperitoneal sarcoma patients treated on ISGSTS0303 protocol. No clinically significant differences were observed among the two methods, even if the supervised one was mainly based on the a priori medical knowledge of the disease. In particular, DFA allowed obtaining a good evaluation of single cases in terms of Mahalanobis distances, which can be possibly converted into probabilities. This methodological approach may well be included into the decision-making process in oncology and, more in general, in medicine, in a sort of “from bed to bioinformatic bench and back” strategy.

## Figures and Tables

**Table 1:** *Distribution of patients by six relevant clinical parameters*. FNCLCC (French Fédération Nationale des Centres de Lutte Contre le Cancer) grading, proliferative index (ki67 expression), and CT-RT preoperative chemo/radiotherapy treatment. For the other acronyms, see the Abbreviations section.

Feature	*N*	%
Age, median (range)	58.5	(27–75)
Sex		
Female	32	45.7
Male	38	54.3
Tumor size (cm), median (range)	15	(5–45)
STS histology		
WDLPS	16	22.9
DDLPS	22	31.4
LMS	15	21.4
Others	17	24.3
FNCLLC grading (missing data = 1)		
G1	19	27.2
G2	32	45.7
G3	18	25.7
Response to CT-RT (missing data = 3)		
SD	50	71.4
PR	8	11.4
PD	9	12.9

**Table 2:** *Clinical descriptors and prognostic reclassification*. Each variable was reclassified according to the prognostic information deriving from literature data and clinical expertise. Each reclassification group could range from 0 (better prognosis) to 3 (worst prognosis). For more details on the recoding, see the Appendix.

Clinical characteristics	Values	Reclassification
Age (years)	<50	1
	51–60	1.5
	61–65	2
	66–70	2.5
	70–75	3

Histology	WDLPS, SFT	1
	Myxoid LPS	1.5
	Pleomorphic LPS, fibrosarcoma	2
	LMS, DDLPS	2.5
	MPNST, NOS sarcoma	3

FNCLLC grading	G1	1
	G2	2
	G3	3

Size (cm)	<10	1
	11–15	1.5
	16–20	2
	20–30	2.5
	30–45	3

Response after chemo- and radiotherapy	PR	1
	SD	2
	PD	3

Type of resection	No resection	1
	R0/R1	2
	R2	3

DFI (months)	Never relapsed	0
	>36	0.5
	24–36	1
	12–24	1.5
	6–12	2
	0–6	2.5
	Never NED	3

Relapse pattern	No relapse	0
	Local	1
	Distant	2
	Local + distant	3

First type of retreatment at recurrence	Surgery, surgery + RT	1
	CT followed by surgery	1.5
	CT or RT	2
	Debulking surgery, no surgery	3

**Table 3:** Univariate DFA over filtered and unfiltered cases. The association with the survival status was independently tested for each of the remaining 9 variables. In the “unfiltered” condition (on the left), the valid cases depended upon the presence of missing cases (to be excluded) on that variable. In the “filtered” condition (on the right), all cases having any type of missing values (9 in total) were excluded.

	Unfiltered					Filtered				
	Valid cases	# wrong	% correct	*R*-squared	−2 log prob.	Valid cases	# wrong	% correct	*R*-squared	−2 log prob.
Age	70	32	54.3	0.00037	96.95	61	27	55.7	−0.003	84.4
Histology	70	27	61.4	0.078	89.40	61	25	59.0	0.060	78.7
Grading	69	25	63.8	0.131	83.01	61	23	62.3	0.114	74.6
Size	68	35	48.5	−0.0023	94.25	61	31	49.2	−0.002	84.28
Response to CTRT	67	26	61.2	0.016	91.38	61	35	42.6	−0.004	84.52
Resection	70	32	54.3	0.02	95.05	61	32	47.5	0.007	83.6
DFI	70	11	84.3	0.49	49.62	61	11	82.0	0.458	45.61
Relapse pattern	66	12	81.8	0.24	69.39	61	11	82.0	0.219	65.71
First type of retreatment	68	15	77.9	0.12	83.36	61	14	77.0	0.120	74.045

**Table 4:** Supervised features of the 70 patients considered in this work. The minus sign indicates cases with missing values, excluded from the analysis.

Patient #	Age	Histology	Grading	Size	CTRT response	Resection	DFI	Relapse pattern	Retreatment (I)	Status
1	3	1	1	2	2	2	0.5	3	1	Alive
2 (−)	1	3	3	1.5	3	1	3		1.5	Dead
3	2	2.5	1	1	2	1	0.5	2	1	Alive
4	2	2.5	2	1	1	1	1.5	1	1	Dead
5	1.5	1	1	2	1	1	0.5	2	1	Dead
6 (−)	1.5	3		2.5	2	1	1.5	1	2	Dead
7 (−)	1	2.5	2	1	2	1	0	0		Alive
8	2.5	3	2	2	2	1	1.5	1	2	Dead
9	1.5	3	2	2.5	2	1	0.5	1	2	Dead
10 (−)	1	2	2	2		1	0.5	1	1	Alive
11 (−)	1.5	3	3		3	3	3		2	Dead
12 (−)	2	1	2	2.5	3	2	3			Dead
13	1.5	1	1	3	2	1	0	0	3	Alive
14	2.5	1	1	2	2	1	1	1	1	Alive
15	1.5	1	2	2	2	1	0	0	3	Alive
16	1.5	1	1	2.5	2	1	0	0	3	Alive
17	3	2	3	1.5	2	1	0	0	3	Alive
18 (−)	2	2.5	2		3	3	3		2	Dead
19	2	3	2	1	3	1	0.5	1	1	Dead
20	1.5	2.5	2	1.5	2	1	1	1	1	Dead
21	1	2.5	3	1.5	2	1	1	1	1	Dead
22	1.5	2.5	3	2.5	2	1	1	1	1	Dead
23	2.5	2.5	2	1	3	1	2	2	2	Dead
24	1.5	3	3	2	2	1	1	1	3	Dead
25	3	2	3	1.5	2	1	1.5	1	3	Dead
26	1.5	2.5	3	1	2	1	1.5	3	3	Dead
27	2	3	3	1	1	1	1.5	1	1	Dead
28	1.5	2.5	3	2	3	1	2.5	3	2	Dead
29	1	2.5	3	3	2	1	0.5	1	2	Dead
30	1.5	1	1	2.5	2	1	0	0	3	Alive
31	1	2.5	3	2	2	1	0	0	3	Dead
32	1.5	1	1	2.5	2	1	0	0	3	Alive
33 (−)	2	1	1	2.5		1	0	0	3	Alive
34	3	2.5	3	1	2	1	1.5	1	2	Dead
35	2	1	3	2	3	1	2.5	2	2	Dead
36	1.5	1	1	2	2	1	0	0	3	Alive
37	2.5	3	2	1.5	2	1	0	0	3	Alive
38	1	2.5	2	1	2	1	0	0	3	Alive
39	1	2	1	1	2	1	0.5	2	2	Dead
40	1.5	1	2	1	1	1	0.5	2	1	Alive
41	1	3	3	1.5	2	1	0	0	3	Alive
42	2.5	2.5	2	1.5	2	1	0	0	3	Alive
43	1	1.5	1	2	1	1	0.5	1	1.5	Dead
44	2.5	2.5	2	2.5	2	1	1.5	1	2	Dead
45	2.5	2.5	2	2.5	2	1	0	0	3	Alive
46	2	2.5	2	2	2	1	0.5	1	2	Dead
47	3	2.5	2	1.5	2	1	1	3	1.5	Dead
48	2	2.5	3	2	2	1	2	3	2	Dead
49	1.5	2.5	2	1	2	1	0.5	1	1.5	Alive
50	1.5	2.5	2	1.5	2	1	0	0	3	Alive
51	2.5	2.5	2	1.5	2	1	2	2	2	Dead
52	1.5	2.5	1	1	2	1	0	0	3	Alive
53	2.5	2.5	2	2.5	2	1	0	0	3	Alive
54	1	2.5	1	2	2	1	0.5	2	2	Alive
55	1.5	2.5	2	1.5	2	1	0	0	3	Alive
56	3	2.5	2	2	1	1	2	1	2	Dead
57 (−)	1.5	2.5	2	2.5		1	1	3	2	Dead
58	2.5	1	1	1.5	2	1	0.5	1	1	Alive
59	2.5	2.5	2	2	2	1	1.5	2	2	Dead
60	1.5	2.5	2	2	2	1	0	0	3	Alive
61	1	1	1	2.5	2	1	0.5	1	1	Alive
62	3	2.5	2	1.5	2	1	0.5	2	2	Alive
63	1.5	1	1	2.5	2	1	0	0	3	Alive
64	2.5	2.5	2	2.5	2	1	0	0	3	Alive
65	1	1	1	3	2	1	0.5	1	2	Dead
66	1	2.5	2	2	2	1	0	0	3	Alive
67	2	1	1	2.5	2	1	0	0	3	Alive
68	2	2.5	3	1.5	3	1	0	0	3	Alive
69	1	2.5	3	1.5	1	1	0	0	3	Alive
70	2.5	2.5	2	2	1	1	1.5	1	2	Alive

**Table 5:** *Multivariate analysis of clinical descriptors by stepwise DFA*. The association with the vital status by stepwise discriminant function analysis (DFA) was carried out according to the casewise (on the left) or the mean-substitution (on the right) methods.

	Casewise	Mean substitution
	Wilks' *λ* (pi level)	Wilks' *λ* (pi level)
DFI	0.60 (<0.001)	0.58 (<0.001)
Histology	0.45 (0.04)	0.43 (0.03)
Age	0.45 (0.04)	0.42 (0.18)
Relapse pattern	0.43 (0.10)	0.43 (0.04)
Size	0.43 (0.17)	0.41 (0.11)

**Table 6:** *Summary of results*. In the casewise method, 4 and 5 cases were excluded because of too many missing values in the supervised and unsupervised methods, respectively. In the mean substitution, no cases were excluded since missing values were substituted by their respective means. Accuracy was computed considering the number of misclassified cases on the total of analyzed cases (68, 67, or 72 in supervised casewise, unsupervised casewise, or mean substitution, resp.).

	Supervised		Unsupervised	
	Casewise	Mean substitution	Casewise	Mean substitution
Incorrect classification	10	11	9	12
Missing values	4	/	5	/
% accuracy	85.3	84.7	86.6	83.3

## Appendix

### Detailed Prognostic Reclassification of Clinical Descriptors in Table 2

Age cut-offs were defined on the basis of the nomogram for retroperitoneal sarcomas [16]. Histology reclassification derived from expertise and specific literature data [22–24]. R0/R1 resection was considered equivalent since there is no radical resection in the retroperitoneal space, by definition. Chemotherapy followed by surgery was reclassified as 1.5 since a chemotherapeutic treatment upfront is usually offered to locally advanced or rapidly growing disease; chemotherapy was evaluated as 2 since it had the objective of controlling the disease but not of eradicating it; no surgery or debulking surgery was classified equally as a 3-point factor (worst prognostic therapeutic approach, since debulking surgery is not effective in RPS and sometimes is also detrimental).

## Abbreviations

3y-RFS: 3-Year relapse-free survival
CT: Chemotherapy
DDLPS: Dedifferentiated liposarcoma
DFA: Discriminant function analysis
DFI: Disease-free interval
DT: Decision trees
FNCLCC: French Fédération Nationale des Centres de Lutte Contre le Cancer
ISG-STS 0303 protocol: Italian Sarcoma Group-Soft Tissue Sarcoma 0303 Protocol
LMS: Leiomyosarcoma
LPS: Liposarcoma
MANOVA: Multivariate analysis of variance
MD: Mahalanobis distance
MPNST: Malignant peripheral nerve sheath tumors
NED: Not evident disease
NOS: Not otherwise specified
OS: Overall survival
PD: Progressive disease
PR: Partial response
R0, R1, and R2 surgical margins: R0, no residual tumor; R1, microscopic residual tumor; R2, macroscopic residual tumor
RECIST: Response Evaluation Criteria in Solid Tumors
Relapse Y/N: Yes/no
RPS: Retroperitoneal sarcoma
RT: Radiation therapy
SD: Stable disease
SFT: Solitary fibrous tumor
STS: Soft tissue sarcomas
WDLPS: Well-differentiated liposarcoma.
